# SENSory re-learning of the UPPer limb after stroke (SENSUPP): study protocol for a pilot randomized controlled trial

**DOI:** 10.1186/s13063-018-2628-1

**Published:** 2018-04-17

**Authors:** Håkan Carlsson, Birgitta Rosén, Hélène Pessah-Rasmussen, Anders Björkman, Christina Brogårdh

**Affiliations:** 1 Department of Neurology and Rehabilitation Medicine, Skåne University Hospital, Lund, Sweden; 2 Department of Health Sciences, Lund University, Lund, Sweden; 3 Department of Translational Medicine—Hand Surgery, Skåne University Hospital, Malmö, Sweden; 4 Department of Clinical Sciences, Lund University, Lund, Sweden

**Keywords:** Stroke, Upper limb, Sensory re-learning, Rehabilitation

## Abstract

**Background:**

Many stroke survivors suffer from sensory impairments of their affected upper limb (UL). Although such impairments can affect the ability to use the UL in everyday activities, very little attention is paid to sensory impairments in stroke rehabilitation. The purpose of this trial is to investigate if sensory re-learning in combination with task-specific training may prove to be more effective than task-specific training alone to improve sensory function of the hand, dexterity, the ability to use the hand in daily activities, perceived participation, and life satisfaction.

**Methods/design:**

This study is a single-blinded pilot randomized controlled trial (RCT) with two treatment arms. The participants will be randomly assigned either to sensory re-learning in combination with task-specific training (sensory group) or to task-specific training only (control group). The training will consist of 2.5 h of group training per session, 2 times per week for 5 weeks. The primary outcome measures to assess sensory function are as follows: Semmes-Weinstein monofilament, Shape/Texture Identification (STI™) test, Fugl-Meyer Assessment—upper extremity (FMA-UE; sensory section), and tactile object identification test. The secondary outcome measures to assess motor function are as follows: Box and Block Test (BBT), mini Sollerman Hand Function Test (mSHFT), Modified Motor Assessment Scale (M-MAS), and Grippit. To assess the ability to use the hand in daily activities, perceived participation, and life satisfaction, the Motor Activity Log (MAL), Canadian Occupational Performance Measure (COPM), Stroke Impact Scale (SIS) participation domain, and Life Satisfaction checklist will be used. Assessments will be performed pre- and post-training and at 3-month follow-up by independent assessors, who are blinded to the participants’ group allocation. At the 3-month follow-up, the participants in the sensory group will also be interviewed about their general experience of the training and how effective they perceived the training.

**Discussion:**

The results from this study can add new knowledge about the effectiveness of sensory re-learning in combination with task-specific training on UL functioning after stroke. If the new training approach proves efficient, the results can provide information on how to design a larger RCT in the future in persons with sensory impairments of the UL after stroke.

**Trial registration:**

ClinicalTrials.gov, NCT03336749. Registered on 8 November 2017.

**Electronic supplementary material:**

The online version of this article (10.1186/s13063-018-2628-1) contains supplementary material, which is available to authorized users.

## Background

Stroke is one of the most common causes of impairments, activity limitations, and participation restrictions in adults [1]. More than half of stroke survivors suffer from sensory impairments of their affected upper limb (UL) [2–4], which are characterized by reduced sense of touch, temperature, proprioception, and pain [2]. The impairments affect the ability to discriminate textures, weights, shapes, and sizes, to grasp and manipulate objects without vision, and to perform bimanual tasks in everyday life [3, 5]. The degree of sensory impairments is associated with stroke severity [2, 6] and recovery of motor performance [7].

The sensory impairments of the UL after stroke often lead to long-term problems to use the UL in daily life [8], such as personal care and household and leisure activities [9, 10]. Despite this, very little attention is paid to sensory impairments in stroke rehabilitation. Instead the focus is on motor recovery, exercises for the lower limbs, and mobility [10, 11]. The possible causes of this discrepancy in sensory and motor rehabilitation are limited knowledge of evidence-based sensory interventions among therapists [12, 13] and lack of use of standardized outcome measures [13].

Different interventions to improve the sensory function are described in the literature, such as mirror therapy, thermal stimulation, and intermittent pneumatic compression [14]. Sensory rehabilitation can be divided into either active sensory training (i.e., manual exploration of different textures, figures, and objects with the hand and fingers, and spatial detection of limb position) or passive sensory training including electrical stimulation, thermal stimulation with hot or cold packs, and pneumatic compression [15, 16]. In a study by Schabrun and Hillier [15], passive sensory training was shown to improve grip strength and dexterity, whereas the effect of active sensory training was unclear. However, other studies have reported positive effects of active sensory training [17, 18]. Carey et al. [19] found that an active sensory discrimination training approach after stroke including texture discrimination, limb position sense, and tactile object recognition (i.e., sensory re-learning training) was more effective than passive sensory training, grasping of common objects, and passive movements of the UL. Furthermore, a few studies have reported that a combination of sensory and motor training including fine motor skills [20], or a learning-based sensorimotor program [21], can improve sensory discrimination and motor control of the UL. However, the studies were based on rather few participants [20, 21] and one study lacked a control group [20].

There is evidence that motor training of the UL in terms of task-specific training with repetitive and context-specific tasks including feedback [22] can improve motor performance of the UL [8, 23]. The sensory and motor systems are closely related [16], and both systems are necessary for accurate and precise movements. To improve overall functioning of the UL after stroke, it may therefore be important to focus not solely on motor training but also on sensory training [24]. However, to the best of our knowledge, no previous study has evaluated the effect of sensory re-learning in combination with task-specific training.

The purpose of this pilot randomized controlled trial (RCT) is therefore to investigate if sensory re-learning in combination with task-specific training may prove to be more effective than task-specific training alone to improve sensation of the hand (primary outcome), dexterity, ability to perform daily hand activities, perceived participation, and life satisfaction (secondary outcomes). The results from this study can provide information on how to design a fully powered RCT in the future.

## Methods/design

### Study design

**Fig. 1:**
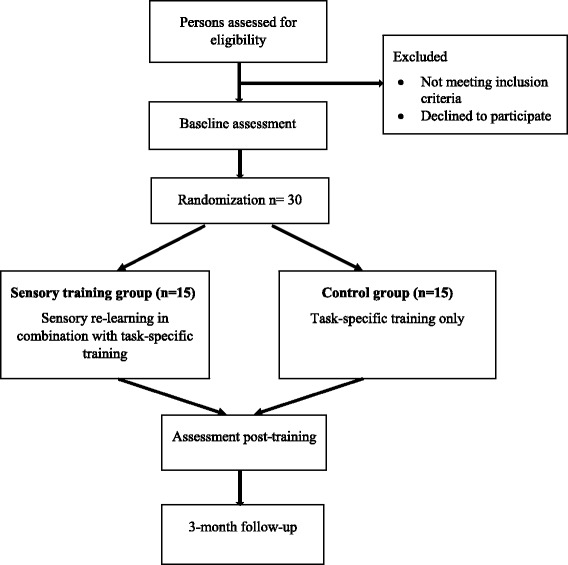
Flow chart of the study design

**Fig. 2:**
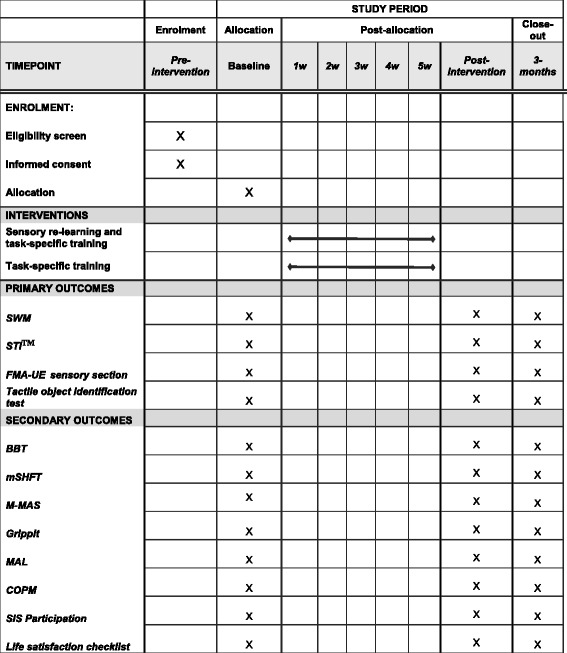
SPIRIT figure of enrollment, interventions, and outcome measures. Abbreviations: *SWM* Semmes-Weinstein monofilament, *STI*^*TM*^ Shape/Texture Identification, *FMA-UE* Fugl-Meyer Assessment—upper extremity, *BBT* Box and Block Test, *mSHFT* mini Sollerman Hand Function Test, *M-MAS* Modified Motor Assessment Scale, *MAL* Motor Activity Log, *COPM* Canadian Occupational Performance Measure, *SIS* Stroke Impact Scale

This is a single-blinded pilot RCT with two treatment arms. Thirty persons with sensory impairments of the UL after stroke will be recruited and randomized either to sensory re-learning in combination with task-specific training (*n* = 15) or to task-specific training only (*n* = 15). In the flow chart (Fig. 1) and the SPIRIT figure (Fig. 2), the overall study design is described, which is in agreement with the SPIRIT checklist (see Additional file 1).

### Inclusion of participants

Potential participants will be identified and recruited by physiotherapists and occupational therapists from the Department of Neurology and Rehabilitation Medicine, Skåne University Hospital or from two outpatient health care settings specialized in stroke rehabilitation. Inclusion criteria are as follows: sensory impairments (≤5 points in the Shape/Texture Identification test [25]) of the UL after stroke, ability to grasp and release an object, ability to understand oral and written information, 18–80 years of age, at least 6 months since stroke onset, and ability to walk with or without an assistive device. Exclusion criteria are as follows: sensory impairments in the UL due to other diagnoses than stroke.

First, a letter with information about the study will be mailed to potential participants. One to 2 weeks later, they will be contacted by phone and asked if they are interested in participating in the study. Additional questions about their sensory-related problems of the UL in daily life will also be asked. Eligible and interested participants will then be assessed at the Department of Neurology and Rehabilitation Medicine.

### Randomization

After the baseline assessments when it has been confirmed that the persons fulfil the inclusion criteria, they will be randomly assigned by sealed paper envelopes either to the sensory group (*n* = 15) or to the control group (*n* = 15) by a person not involved in the training.

### Interventions

The training will consist of 2.5 h of group training with two to three participants per session, twice a week for 5 weeks. Each training session will be supervised by one of the two physiotherapists involved in the study, who are experienced in stroke rehabilitation.

### Training for the sensory group

The design of the sensory re-learning is influenced by Carey et al. [19], Yekutiel and Guttman [17], and Rosén and Lundborg [26]. Sensory re-learning is the patient-oriented expression of sensory re-education which traditionally has been used to describe the concept as: “the gradual and progressive process of reprogramming the brain through the use of cognitive learning techniques such as visualization and verbalization, the use of alternate senses such as vision or hearing and the use of graded tactile stimuli designed to maintain and/or restore sensory areas affected by nerve disorder to improve tactile gnosis” [27].

Each training session will consist of 1-h sensory re-learning comprising three 20-min sessions of (1) touch detection to explore different surfaces; (2) touch discrimination to identify different materials, shapes, textures, weights, and temperatures; and (3) tactile object recognition to examine and identify different objects and proprioception. After a 15-min break, the participants will continue with 1 h of task-specific training comprising three 20-min sessions of (1) tying shoelaces, doing buttons, and pulling up a zipper; (2) fine motor training and bimanual tasks such as pouring water into a bottle and using cutlery; and (3) shuffling, dealing, and turning cards and playing board games.

All participants will be informed about the sensory re-learning approach, i.e., to perform repetitive, graded exercises with increased difficulty, attentive exploration of the stimuli, prevention of vision, and continuous feedback of the impaired sensation via vision or the unaffected hand. They are encouraged to reflect on their sensation when having an object in their affected hand, about the characteristics of the object, size, texture, material, and weight. When they are using their affected hand in daily activities, they should also carefully think of the object’s properties. Individualized home exercises once a week will also be performed including follow-ups of the home activities.

### Training for the control group

The training for the control group includes task-specific exercises without any focus on sensory re-learning. The task-specific training will consist of 2-h practicing comprising six 20-min sessions (including a 15-min break) with exercises of (1) daily activities such as tying shoelaces, doing buttons, and pulling up a zipper; (2) fine motor training and bimanual tasks such as pouring water into a bottle and using cutlery; (3) shuffling, dealing, and turning cards and playing board games; but also (4) strength training for the UL with a Theraband; (5) active arm movements such as reaching for objects; and (6) stretching of the UL. The participants will also be encouraged to use their affected UL as much as possible in daily activities at home.

### Outcome measures and assessors

To be able to evaluate the effects of interventions, outcome measures with good psychometric properties will be used, covering different domains of the International Classification of Functioning, Disability and Health (ICF) [28]. Two occupational therapists, who are blinded to the group allocation and with long experience of stroke rehabilitation and the outcome measures used, will conduct all assessments pre- and post-training and at 3-month follow-up. At the 3-month follow-up, the participants in the sensory training group will also be interviewed regarding their experiences and perceived effects of the training by an interviewer not involved in the training.

### Primary outcome measures

#### Semmes-Weinstein monofilament

This is to assess touch detection thresholds [29] of the hand and fingers. The short version (pocket filaments) with five filaments from 0.07 to 279 g will be used (Touch Test® Sensory Evaluators, North Coast Medical Inc.). The touch detection thresholds are scored on a 0 to 5-point scale, where 5 represents the thinnest filament and 0 represents the largest filament. Five different positions of the hand are tested: the fingertip on digits I, II, and V and the thenar and hypothenar regions, yielding a total sum score of 25 points. The pocket filaments have been used previously in stroke studies [30].

#### Shape/Texture Identification (STI™) test

This is to measure the ability to identify shapes (cube, cylinder, and hexagon) and textures (one, two, or three dots in a row) in decreasing sizes [31] (www.sensory-test.com). The scores range from 0 to 3 points per hand for each subtest with a maximum score of 6 points. STI™ has been shown to have high test-retest reliability in persons with mild to moderate disability after stroke [25].

#### Fugl-Meyer Assessment—upper extremity (FMA-UE; sensory section)

This is to measure light touch and proprioception of the UL after stroke [32]. The score ranges from 0 to 4 points for each subtest with a maximum score of 8 points per hand. It has been shown to be a clinically useful and a robust instrument in persons with sensory impairments after stroke [33].

#### Tactile object identification test

This is to measure the ability to identify different objects without vision. The test is based on the original test by Yekutiel and Guttman [17]. Out of 20 objects, 15 are used during the assessment. Within 15 s, the participant should blind-folded recognize an object. A correct answer yields 2 points, recognition of some features of the object yields 1 point, and an incorrect answer yields 0 point, yielding a maximum total sum score of 30 points.

### Secondary outcome measures

#### Box and Block Test (BBT)

This is to assess gross manual dexterity. It consists of a box with two compartments and of 100 wooden blocks. The number of blocks that can be transported from one compartment to the other during 1 min is counted [34]. The BBT has been shown to be reliable in persons with mild to moderate disability after stroke [35].

#### The mini Sollerman Hand Function Test (mSHFT)

This is to assess fine manual dexterity (PROcare ApS, www.procare.dk). It consists of three selected tasks from the original Sollerman test of 20 items [36]: (1) picking up four coins from a purse, (2) putting four nuts on bolts, and (3) buttoning four buttons. The score ranges from 0 to 4 points for each task with a maximum score of 12 points. The mSHFT has been shown to be reliable in persons with mild to moderate disability after stroke [35].

#### Modified Motor Assessment Scale (M-MAS) of the UL

This is to assess dexterity by five tasks in the advanced hand activity domain. The scale ranges from 0 to 5 points, where 0 represents no motor function and 5 represents almost normal or normal motor function. The M-MAS is a further development of the Motor Assessment Scale [37] and has been shown to have good reliability and validity after stroke [38].

#### Grippit

This is to measure isometric grip strength using a computerized wireless dynamometer (http://www.catell.se). The highest isometric contraction of three trials is recorded in newtons (N). The Grippit has been shown to be reliable in persons with mild to moderate disability after stroke [39].

#### Motor Activity Log (MAL)

This is to assess the participants’ perception of how much (amount of use (AOU)) and how well (quality of movement (QOM)) they use their affected hand in daily activities. The MAL consists of 30 items where the response options range from 0 to 5 for both AOU and QOM, each yielding a score of 150 points. The MAL has been shown to be reliable after stroke [40].

#### Canadian Occupational Performance Measure (COPM)

This is to identify the participants’ problems in their execution of activities in self-care, productivity, and leisure activities [41]. The self-perceived performance and satisfaction of their sensory-related problems are rated on a scale ranging from 1 (do not perform well and not satisfied) to 10 (perform extremely well and extremely satisfied). COPM has shown moderate to good test-retest reliability in persons with stroke [42].

#### Stroke Impact Scale (SIS) participation domain

This is to assess the participants’ perceived participation. The domain consists of eight items, where each item ranges from 5 (limited none of the time) to 1 (limited all of the time). The mean value for the items is calculated and converted into a percentage value from 0 to 100. SIS has shown to be reliable, valid, and sensitive to change in persons with mild to moderate stroke [43].

#### Life Satisfaction checklist

This is to measure perceived life satisfaction by one global item (“Life as a whole”) and ten domain-specific items [44]; in this study, only the global item will be used. The rating ranges from 1 (very dissatisfied) to 6 (very satisfied).

### Data analysis

Descriptive statistics (mean (SD) or median (minimum–maximum)) will be used to characterize the study groups. As this is a new treatment approach, no power calculation can be performed. However, 30 participants can be considered sufficient to include in a pilot RCT within a reasonable time frame. To analyze potential differences between the groups, the Mann-Whitney test (for ordinal data) or independent sample *t* test (for continuous data) will be used. The Wilcoxon signed-rank test or paired *t* test will be used to analyze within-group differences. The level of statistical significance will be set at *p* ≤ 0.05. All calculations will be performed using IBM SPSS Statistics version 22 (IBM Corporation, Armonk, NY, USA).

The interviews of the participants in the sensory group at the 3-month follow-up will be recorded and transcribed verbatim. Data will be analyzed with an inductive content analysis approach [45].

### Withdrawal and safety

The participants may withdraw from the study at any time without giving an explanation and without any negative consequences for their care or rehabilitation in the future. Based on previous studies of sensory re-learning [19], our judgement is that there is a low risk of adverse events in the SENSUPP study.

### Data management

Data from all assessments before and after the intervention and at the 3-month follow-up will be decoded and stored in a database protected by a password to which only researchers responsible have access. All files will be saved for at least 10 years after completion of the study. The data will be disseminated by publication in scientific journals.

## Discussion

The SENSUPP study is a pilot RCT with two treatment arms. The purpose is to investigate if sensory re-learning in combination with task-specific training may prove to be more effective than task-specific training alone to improve sensory function of the hand, dexterity, the ability to perform daily hand activities, perceived participation, and life satisfaction in patients with stroke and residual sensory impairments in the UL. As the sensory and motor systems are closely related [16], it may be important to focus not solely on task-specific training but also to include sensory training in the rehabilitation of the UL after stroke.

Previous studies have primarily evaluated the effect of different sensory training approaches on sensory function [17, 19, 21] but not if the training has any effect on activity and participation. To be able to evaluate the effect of sensory re-learning training on daily life, it is important to use psychometrically sound outcome measures according to the International Classification of Functioning, Disability and Health (ICF) [46], as will be used in the present study.

The results from the SENSUPP study will add new knowledge about the feasibility and effectiveness of sensory re-learning in combination with task-specific training on UL functioning after stroke. It may also contribute to an increased understanding of how the participants perceive the sensory training of the affected hand and the possible effects of training. If the new training approach proves efficient, results from the SENSUPP study can provide knowledge on how to design a larger RCT in persons with sensory impairments of the UL after stroke.

### Trial status

The recruitment of participants started 1 April 2017, and the trial is expected to continue until December 2019.

## Additional file

Additional file 1: SPIRIT checklist. (DOC 120 kb)

## Abbreviations

BBT: Box and Block Test
COPM: Canadian Occupational Performance Measure
FMA-UE: Fugl-Meyer Assessment—Upper Extremity
ICF: International Classification of Functioning, Disability and Health
MAL: Motor Activity Log
M-MAS: Modified Motor Assessment Scale
RCT: Randomized Controlled Trial
SIS: Stroke Impact Scale
STI™: Shape/Texture Identification
UL: Upper Limb
